# Supplementary material for: A Humanized Yeast Phenomic Model of Deoxycytidine Kinase to Predict Genetic Buffering of Nucleoside Analog Cytotoxicity
Source: Genes (Basel). 2019 Sep 30;10(10):770. doi: 10.3390/genes10100770 (PMC6826991; doi:10.3390/genes10100770)
Supplement: Supplementary file 1 [file genes-10-00770-s001.zip › Additional_Files/Additional_File7_GOTermHeatmaps.zip!/Additional_File7_GOTermHeatmaps/Elongator_holoenzyme_complex.pdf]

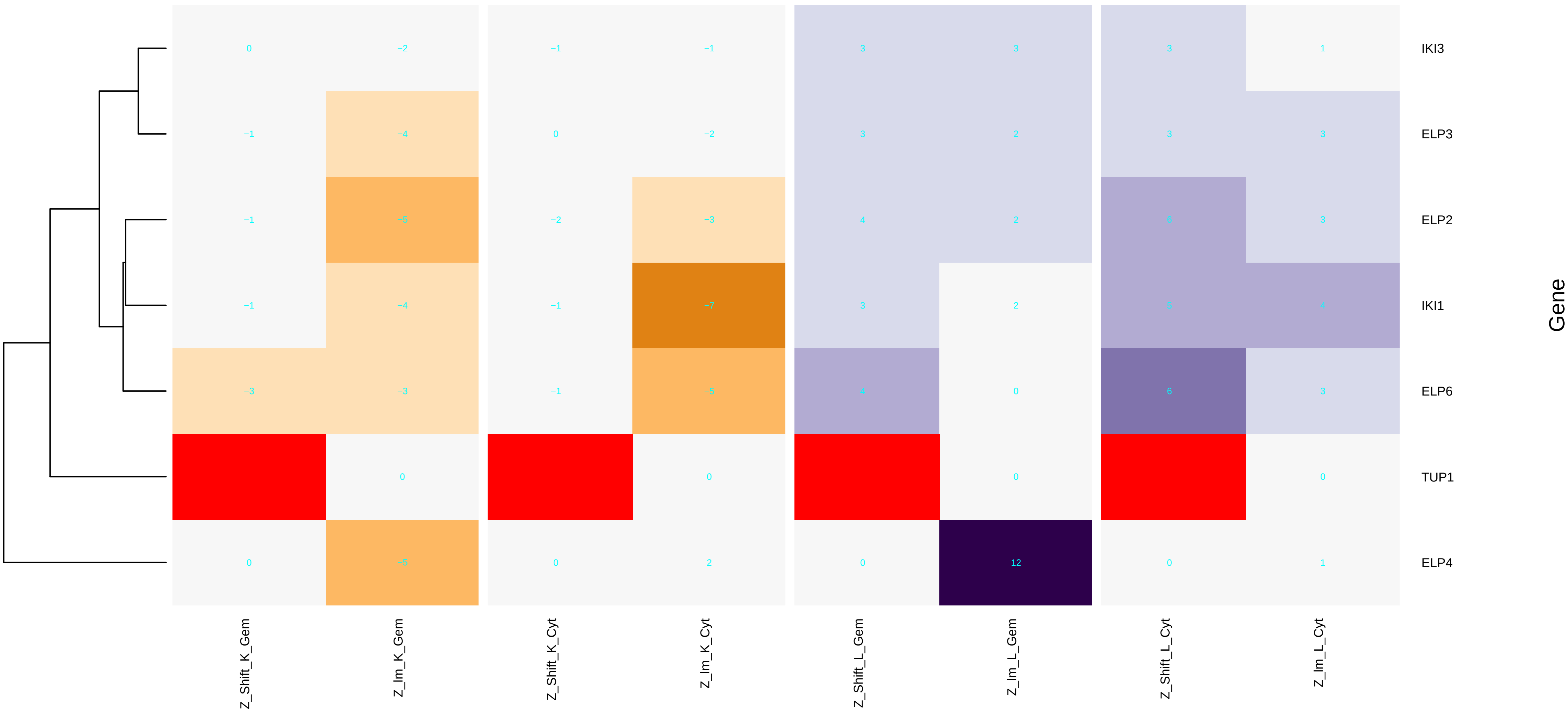

IKI3
ELP3
ELP2
IKI1
ELP6
TUP1
ELP4
Gene
Z_Shift_K_Gem
Z_Im_K_Gem
Z_Shift_K_Cyt
Z_Im_K_Cyt
Z_Shift_L_Gem
Z_Im_L_Gem
Z_Shift_L_Cyt
Z_Im_L_Cyt
0 −2 −1 −1 3 3 3 1
−1 −4 0 −2 3 2 3 3
−1 −5 −2 −3 4 2 6 3
−1 −4 −1 −7 3 2 5 4
−3 −3 −1 −5 4 0 6 3
0 0 0 0
0 −5 0 2 0 12 0 1
